# Exploring the Effectiveness of Emergency Medical Services Becoming Active in Fall Prevention: A Literature Review

**DOI:** 10.7759/cureus.61541

**Published:** 2024-06-02

**Authors:** Luke Speier, Nicholas Kramer, Varna Jammula, Sydney Kramer, Graal Diaz

**Affiliations:** 1 Osteopathic Medicine, Touro University Nevada, Henderson, USA; 2 Occupational Therapy, Touro University Nevada, Henderson, USA; 3 Research and Development, Ventura County Medical Center, Ventura, USA

**Keywords:** community paramedicine, first responders, fall risk, emergency medical services, fall prevention

## Abstract

Falls, particularly among the elderly, are a prevalent and growing healthcare issue in the United States. Individuals who experience falls face heightened morbidity and mortality risks, along with substantial expenses associated with managing any resulting injuries. First responders frequently respond to 911 calls related to falls, with a significant portion of these cases not resulting in hospital or healthcare facility transfers. As such, many fall victims receive treatment without any preventive measures being implemented. The purpose of this review is to explore the current studies that examine whether Emergency Medical Service personnel can effectively act in fall prevention. While earlier studies present conflicting findings, recent research indicates the potential for preventive strategies that go beyond mere referrals.

## Introduction and background

In the United States, 25% of senior citizens fall every year, and their risk increases as they age [1,2]. As of 2020, there were about 56 million senior citizens in the United States; by 2040, that number is expected to rise to over 80 million [3]. Therefore, falls among the elderly represent a growing public health issue. Nationwide, falls lead to medical costs totaling over $50 billion per year and are the leading cause of Emergency Department (ED) visits and death in people over the age of 65 [4-6]. The average hospital visit cost due to a fall is over $29,000 [7]. Additionally, there are unmeasurable costs; as after a fall, the patient may have trouble with activities of daily living, requiring a family member or friend to skip work to provide care. For falls among senior citizens, about one in five results in a severe injury such as a fracture or head trauma [8]. Ninety-eight percent of hip fractures occur due to falling, often requiring surgery and extensive recovery time [9,10]. Falls can be a harbinger of decline beyond injury; in 2009, a study revealed that just two falls by an elderly person are associated with increased mortality [11]. Falls are often a recurring problem for senior citizens; data indicates that after one fall, the chances of experiencing a second nearly triple [12]. Considering this information, healthcare providers are eager to create and investigate preventative measures.

A multidisciplinary strategy is crucial for fall prevention. Occupational therapy (OT) and physical therapy (PT) offer vital services that have effectively reduced the likelihood of falls. These services encompass environmental modifications, targeted exercises to enhance strength, range of motion, endurance, balance and gait, managing medications, addressing postural hypotension, suggesting suitable footwear, and addressing psychological factors such as fear of falling [13]. Research indicates that an individual's fear of falling negatively affects activity performance, increasing their risk of falling [14,15]. Hence, OTs play a unique role in improving the self-efficacy and confidence of older adults in carrying out their daily activities [16,17]. Furthermore, current literature suggests that multicomponent prevention programs have reduced the risk of falls. A comprehensive fall prevention program provided in an inpatient rehab facility across the disciplines of nursing, PT, and OT demonstrated significantly fewer falls than those who did not participate in the program. This program included an informational brochure, exercise routines, educational sessions, and the use of hip protectors [18].

A randomized control trial where individuals completed exercise sessions three times a week for 45 minutes in addition to usual care had a statistically significant lower incidence of falls than those who only received usual care [19]. Another study demonstrated that older adults who participated in home health visits with individually tailored exercise programs three times a week for 30 minutes and walking twice a week for a year experienced fewer falls than those in the control group [20]. This research highlights the positive impact of integrating exercise into one's daily routine and its effects on one's occupational performance and quality of life. A recent study of post-acute fall prevention demonstrated that the implementation of a brief, tailored consultation from a physical therapist and pharmacist during a post-fall emergency visit reduced six-month ED encounters [21]. Lastly, multiple studies have determined the effectiveness of physical activity in reducing falls. Exercises aimed at aerobic training, balance, and strengthening are most effective in lowering the rate of falls [22]. Likewise, exercise as a single intervention reduced the number of falls and recurrent falls by 36% and 41%, respectively, in people living in nursing homes [23]. Lastly, a group of researchers conducted an assessment of the risk of falls related to the Beers Criteria for Potentially Inappropriate Medication (PIM) and polypharmacy. They found that among elderly patients, 55% had a high risk of falling, and 21% of patients had fallen during the past year. Furthermore, the researchers found that polypharmacy was prevalent in 50.90% of all patients, whereas the Beers criteria PIM-positive group accounted for 51.42% of the study population [24].

In general, the existing body of literature demonstrates the vital role of multidisciplinary healthcare in preventing falls and the various interventions employed to enhance occupational performance, improve quality of life, and ultimately decrease the financial burden of healthcare costs associated with falls. Emergency Medical Service (EMS) personnel are often the first responders in treating adults who have fallen, but sometimes transfer does not occur; up to 21% of falls in some communities do not result in transport to a healthcare facility; these are deemed “lift-assist calls.” Unfortunately, 23% of patients who are lift-assisted are later found to have a traumatic injury when seen by a provider. Lift-assist calls take up a significant portion of an EMS call quota, as much as 17% [25]. This data suggests that EMS personnel are often the only healthcare professionals to treat a fall victim. Few fall prevention programs exist for patients treated outside of clinics, hospitals, or other healthcare facilities. This poses a problem as lift-assist calls are associated with increased morbidity and mortality. Within just two weeks, 21% of these calls result in an emergency department (ED) visit, 12% result in hospitalization, and 1% of patients die. More than one vital sign not being taken at a lift-assist call is correlated with an increased likelihood of hospital admission [26]. Another study revealed that up to 50% of lift-assist calls result in a second 911 call for a fall within the span of a month [27].

There has been a recent push to expand the role of EMS in healthcare. Many EMS groups are pushing toward Community Paramedicine/Mobile Integrated Health (CP/MIH), where EMS personnel are involved in preventive services in addition to their usual role in acute care [28]. A 2017 study by Agarwal and colleagues explored the effectiveness of this change [29]. EMS personnel regularly visited a senior citizen community for one year to take vitals and blood glucose levels, reducing the risk of hypertension and diabetes. Furthermore, 911 calls were decreased by 25%. Although this study is somewhat unrealistic, given that attentive services are provided at no cost to patients, it does show that EMS personnel can successfully act in preventative healthcare [29].

## Review

Given the potential effectiveness of first responders becoming active in preventative medicine, we herein review the literature on the potential for fall reduction. PubMed and Google Scholar were searched using the terms "first responder", "fall prevention", "EMS/emergency medical services", "lift assist", "lift assist call", “paramedics”, and “ambulance”. No time restraint was implemented within our search. Studies were included in this review based on the following criteria: English-language articles, involved a pre-hospital emergency response team, used a qualitative or quantitative fall-risk assessment, and participants with an average age of 60 years and older. Studies were excluded if no risk of falls was assessed and the intervention did not take place in a pre-hospital setting. Ten articles met our inclusion criteria.

Overall, studies have yielded mixed results. In a 2010 study in the United Kingdom, adults in a region over the age of 60 who experienced a lift-assist call were referred to a fall prevention program. Notably, the paramedics responding to the call did not orchestrate the referral, but the participants were identified by 911 call records and visited by a person conducting the study. The fall prevention program consisted of in-home nursing and occupational therapy visits and classes at a community center run by physiotherapists. Participants had the option to choose up to 12 classes of in-home or community-based activities. The control group consisted of lift-assist callers in the area who received standard medical care without a referral. The study was run for 12 months, and the incidence rate ratio of falls per year in the intervention group was statistically significant at 0.45. Participants in the study completed an average of 9.9 sessions over the course of the study [30]. An aspect of the study that may have yielded better results is the pressure taken off of paramedics to make referrals. One of the problems with this study is that the intervention and control groups consisted of only 102 participants each, and falls were recorded by participants in a diary, subjecting the study to recall bias. A similar study was conducted in 2017, in which paramedics referred fall victims over the age of 65 to a community-based fall prevention program. The control group consisted of fall victims in the same age demographic who did not receive a referral. Emergency healthcare contacts, including ED visits, ED admissions, and EMS calls, were recorded at one and six months post-fall. Overall, no statistically significant difference was found between the control and intervention groups in total emergency healthcare contacts. There was a small but significant reduction in EMS calls specifically, with an absolute risk reduction of 3.3. Despite the relatively short outcome time of six months, this study had a sample size of over 2,300 for both the control and intervention groups [31]. Another study involved adults 65 and older who received fall-related paramedic care but were not transported to the emergency department. Paramedics identified participants following the non-transportation to the hospital, and these participants were randomized into an intervention or control group. Those in the intervention group received referrals for exercise therapy, home hazard assessments by occupational therapy, visual assessments by optometrists, and medication reviews by pharmacologists and hospital outpatient clinics. Participants in the control group only received written advice on speaking with their providers and addressing their fall risk factors. During a 12-month follow-up period, this study found no significant difference in the number of falls between the groups. Those in the intervention group who completed all the recommended interventions did have a significantly lower fall rate than those who did not complete the interventions. However, this difference was not statistically significant when comparing these individuals to the control group [32]. Therefore, a certain amount of doubt is cast on the effectiveness of EMS intervention in fall prevention.

A critical lens through which to examine this subject through is whether first responders have time to refer patients. In a 2013 study in Australia, paramedics were trained extensively to refer lift-assist victims 65 and older to an eight-week community-based fall prevention program. An electronic consent form was used to facilitate the referral process. The control group consisted of people over the age of 65 who were referred to the same fall prevention service by their primary care provider (PCP) or a hospital. In the intervention group, paramedics referred only 2.6% of eligible patients to the fall prevention program [33].

Additionally, more people in the control group received the referral and completed the fall prevention program [33]. This begs the question of whether first responders have the time or resources to discuss preventive services with patients. However, a similar study in the United States in 2016 by Phelan and colleagues revealed more success. The population was individuals aged 65 and older who called 911 for a fall. EMS providers provided a sheet containing information about two fall prevention programs to patients who met the specified criteria. One program was offered at a fall assessment clinic, while the other involved in-home visits. Data from a neighboring fire department with a similar call volume served as the control. The study found that 49% of fall victims were given a referral sheet. In post-study interviews, EMS personnel noted the referral task did not have a significant toll on their workload. It is worth mentioning that fall victims had a poor follow-up rate with the fall prevention program. Only 6% of people who received a referral enrolled in a fall prevention program, compared to 3% of the control group [34].

Two studies have reported on the feasibility of screening and referral by EMS providers in rural communities in New York, USA [35,36]. One study involved two rural communities with adults above the age of 65 years who received EMS care. Emergency Medical Technicians (EMTs) received monthly training on how to ask screening questions, provide care for older adults, and evaluate the home environment. The screening questions identified those at risk for falls and infections. EMTs provided patients with educational materials, and the information about their status was sent to their PCP. The control group consisted of adults above the age of 65 years who received usual EMS care and were not asked screening questions. Eighty percent of patients in the intervention group were successfully screened by EMS, indicating that this program was feasible. However, only 21% of patients could recollect the educational materials about falls provided to them, and only 10% discussed falls with their PCP [35]. While the program may be feasible, a more involved intervention may be necessary to ensure the patient recalls educational information about fall prevention and follows up with their PCP. The other study involved EMS referral to community-based case managers for in-home evaluations and referral to resources. Adults above the age of 60 who received EMS care were screened by EMS providers via questionnaires for falls, depression, and medication management. These providers then referred patients to case managers for an in-home evaluation, including falls and home environmental safety. After the evaluation, the case manager made direct referrals to community resources, including the patient’s PCP. EMS screened 85% of patients, and of the patients who completed an in-home assessment, 46% had a fall-related environmental risk, and 54% had a fall during the past year [36]. Although patient outcomes were not reported, this study also supports the feasibility of EMS in screening for the risk of falls and providing this new information to the patient’s PCP.

Despite some evidence calling into question how effective EMS can be in fall prevention, two recent studies have shown very promising results [37,38]. The 2022 study by Quatman-Yates et al. established a Community Paramedicine/Mobile Integrated Health (CP/MIH) program in a midwestern city in the United States that identified people potentially at high risk for falls through 911 calls, self-referrals, or referrals by a physician. During 911 calls, EMS personnel could make referrals to the CP/MIH team, but the CP/MIH team also reviewed 911 records to look for high-risk patients. Once candidates were identified, the CP/MIH team came to the patient's house for an extensive assessment. They examined medications, the living environment, and took vitals. Grab bars were placed as needed, and necessary referrals were placed to various providers. Notably, the Stopping Elderly Accidents, Deaths and Injuries (STEADI) clinical pathway from the Centers for Disease Control and Prevention (CDC) [39] and the Home Safety Self-Assessment Tool (HSSAT) [40] were followed to orchestrate the intervention. The study consisted of four phases. An exploration phase of 12 months of no CP/MIH team served as the control, followed by a preparation phase where the CP/MIH team was active but not in fall prevention specifically. Then came an implementation phase involving a plan-do-study act, and lastly, a 12-month sustainment phase where the CP/MIH fall intervention team processes were fully refined. The CP/MIH team conducted 517 home visits during the sustainment phase, with 109 new visits and 398 follow-ups. The outcome compared the sustainment phase to the exploration phase, with a statistically significant relative risk reduction for total falls and 911 calls for falls at 0.63 and 0.66, respectively. Additionally, the relative risk reduction for fall-related calls resulting in transport was statistically significant at 0.49 [37]. One limitation of this study was that it received substantial financial contributions from first responder services, so it is unclear if it was cost-effective overall. In 2024, Camp and colleagues [38] performed a four-year prospective cohort study similar to the study by Quatman-Yates et al. In a large metropolitan area in the United States, paramedics were tasked to identify 911 callers 65 and older at risk for falls and refer them to a Mobile Integrated Health (MIH) group run by EMS. The STEADI Stay Independent questionnaire [41] was used to screen patients. Additionally, patients at high risk for falling were referred to the MIH by local hospitals upon discharge. Those identified by 911 calls were labeled the High Utilization Group (HUG), and the referrals from hospitals made up the Hospital Readmission Avoidance (HRA) program. For the intervention in both the HUG and HRA programs, the MIH group visited patients' homes and provided preventative services guided by the STEADI clinical pathway, including review of medications, necessary referrals, and adjustments to the home environment. In the HUG, visits were offered up to two times a week for 12 weeks, and in the HRA group, visits were offered twice a week for four weeks. EMS data from the year before the intervention was implemented served as the control. Ultimately, 45,090 individuals aged 65 and older were screened, and 59.3% were at risk for falls. The HUG and HRA programs combined resulted in a 37% reduction in the rate of fall-related 911 calls in the community and a 13% decrease in hospital readmission rates for admitted fall victims. For the HUG, the intervention resulted in savings of over $19,000 per patient and $1 million overall, with the HRA group eclipsing that at a total savings of $4.95 million. A limitation of this study, which is noted by the authors, is that this model would only be possible in areas strongly staffed with EMS personnel given the extensive intervention implemented [38]. There are promising results here, but integrating this into the US healthcare system would require collaboration from EMS, insurance companies, and other healthcare entities. The reason that these two studies mentioned here showed promising results is likely because they involved extensive levels of intervention. In both studies, CP/MIH teams visited the homes of high-risk fallers and followed STEADI criteria. This suggests that for EMS to prevent falls, more needs to be done other than just making a referral to an outside fall prevention program.

Further studies need to be done to clarify the effectiveness of EMS acting in fall prevention. An aspect of this possibility that remains unclear is the cost of hours logged by CP/MIH teams during home visits. This would need to be tested further in collaboration with medical insurance companies to determine overall cost-effectiveness. One study examined the cost-effectiveness of implementing a community fall prevention service [42] through the protocol outlined by Logan et al. [30]. Sach et al. (2010) reported the intervention group had, on average, 5.34 fewer falls over 12 months when compared to the control group. Subsequently, the intervention group had fewer visits to their primary care providers, less in-patient or social service care, and less ambulance service use. The results of this study suggest that the implementation of a fall prevention service is cost-effective and improves patient outcomes [42]. However, this study was done in the United Kingdom, which has a publicly funded healthcare system. While the results seem promising, understanding the cost-effectiveness of these programs in the US requires cooperation with various organizations, including insurance companies. Another area to be tested further is how EMS personnel in rural communities with limited resources could act in fall prevention. An EMS group with minimal staffing would unlikely be able to provide extensive intervention. It would be prudent to explore if quick home check-ins and brief monitoring in line with STEADI criteria would be effective for high-risk fallers.

## Conclusions

Given the relationship of falls to cost and re-injury, widespread prevention methods are indeed indicated. So far, the studies on EMS becoming active in fall prevention have yielded mixed results. A pattern has arisen where more extensive intervention from EMS has been efficacious. More research needs to be conducted on this matter to explore its efficacy in different settings, feasibility, and cost-effectiveness.

## References

[1] Bergen G, Stevens MR, Burns ER (2016). Falls and fall injuries among adults aged ≥65 years: United States, 2014. MMWR Morb Mortal Wkly Rep.

[2] Alshammari SA, Alhassan AM, Aldawsari MA, Bazuhair FO, Alotaibi FK, Aldakhil AA, Abdulfattah FW (2018). Falls among elderly and its relation with their health problems and surrounding environmental factors in Riyadh. J Family Community Med.

[3] (2024). Vespa J, Medina L, Armstrong DM: Demographic turning points for the United States: population projections for 2020 to 2060. Demographic Turning Points for the United States: Population Projections for.

[4] Burns E, Kakara R (2018). Deaths from falls among persons aged ≥65 years: United States, 2007-2016. MMWR Morb Mortal Wkly Rep.

[5] Moreland B, Lee R (2021). Emergency department visits and hospitalizations for selected nonfatal injuries among adults aged ≥65 years: United States, 2018. MMWR Morb Mortal Wkly Rep.

[6] Florence CS, Bergen G, Atherly A, Burns E, Stevens J, Drake C (2018). Medical costs of fatal and nonfatal falls in older adults. J Am Geriatr Soc.

[7] Su FY, Fu ML, Zhao QH, Huang HH, Luo D, Xiao MZ (2021). Analysis of hospitalization costs related to fall injuries in elderly patients. World J Clin Cases.

[8] Vaishya R, Vaish A (2020). Falls in older adults are serious. Indian J Orthop.

[9] Parkkari J, Kannus P, Palvanen M (1999). Majority of hip fractures occur as a result of a fall and impact on the greater trochanter of the femur: a prospective controlled hip fracture study with 206 consecutive patients. Calcif Tissue Int.

[10] Schnell S, Friedman SM, Mendelson DA, Bingham KW, Kates SL (2010). The 1-year mortality of patients treated in a hip fracture program for elders. Geriatr Orthop Surg Rehabil.

[11] Sylliaas H, Idland G, Sandvik L, Forsen L, Bergland A (2009). Does mortality of the aged increase with the number of falls? Results from a nine-year follow-up study. Eur J Epidemiol.

[12] Tinetti ME, Speechley M, Ginter SF (1988). Risk factors for falls among elderly persons living in the community. N Engl J Med.

[13] Leland NE, Elliott SJ, O'Malley L, Murphy SL (2012). Occupational therapy in fall prevention: current evidence and future directions. Am J Occup Ther.

[14] Walker JE, Howland J (1991). Falls and fear of falling among elderly persons living in the community: occupational therapy interventions. Am J Occup Ther.

[15] Zijlstra GA, van Haastregt JC, Ambergen T, van Rossum E, van Eijk JT, Tennstedt SL, Kempen GI. (2009). Effects of a multicomponent cognitive behavioral group intervention on fear of falling and activity avoidance in community-dwelling older adults: Results of a randomized controlled trial. J Am Geriatr Soc.

[16] Cheal B, Clemson L (2001). Older people enhancing self‐efficacy in fall‐risk situations. Aust Occup Ther J.

[17] Peterson EW, Murphy S (2002). Fear of falling: Part II. Assessment and intervention. Home and Community Health Special Interest Section.

[18] Haines TP, Bennell KL, Osborne RH, Hill KD (2004). Effectiveness of targeted falls prevention programme in subacute hospital setting: randomised controlled trial. BMJ.

[19] Haines TP, Hill KD, Bennell KL, Osborne RH (2007). Additional exercise for older subacute hospital inpatients to prevent falls: benefits and barriers to implementation and evaluation. Clin Rehabil.

[20] Robertson MC, Devlin N, Gardner MM, Campbell AJ (2001). Effectiveness and economic evaluation of a nurse delivered home exercise programme to prevent falls. 1: Randomised controlled trial. BMJ.

[21] Goldberg EM, Marks SJ, Resnik LJ, Long S, Mellott H, Merchant RC (2020). Can an emergency department-initiated intervention prevent subsequent falls and health care use in older adults? A randomized controlled trial. Ann Emerg Med.

[22] (2024). US Department of Health and Human Services: Physical Activity Guidelines for Americans Second Edition. https://health.gov/sites/default/files/2019-09/Physical_Activity_Guidelines_2nd_edition.pdf.

[23] Gulka HJ, Patel V, Arora T, McArthur C, Iaboni A (2020). Efficacy and generalizability of falls prevention interventions in nursing homes: a systematic review and meta-analysis. J Am Med Dir Assoc.

[24] Walker BS, Collier BR, Bower KL (2019). The prevalence of Beers Criteria Medication Use and associations with falls in geriatric patients at a Level 1 trauma center. Am Surg.

[25] Faul M, Stevens JA, Sasser SM, Alee L, Deokar AJ, Kuhls DA, Burke PA (2016). Older adult falls seen by Emergency Medical Service providers: a prevention opportunity. Am J Prev Med.

[26] Leggatt L, Van Aarsen K, Columbus M, Dukelow A, Lewell M, Davis M, McLeod S (2017). Morbidity and mortality associated with prehospital "lift-assist" calls. Prehosp Emerg Care.

[27] Cone DC, Ahern J, Lee CH, Baker D, Murphy T, Bogucki S (2013). A descriptive study of the "lift-assist" call. Prehosp Emerg Care.

[28] (2024). EMS.gov: Planning for the future: EMS Agenda 2050. https://www.ems.gov/issues/planning-for-the-future-ems-agenda-2050/.

[29] Agarwal G, Angeles R, Pirrie M, Marzanek F, McLeod B, Parascandalo J, Dolovich L (2017). Effectiveness of a community paramedic-led health assessment and education initiative in a seniors' residence building: the Community Health Assessment Program through Emergency Medical Services (CHAP-EMS). BMC Emerg Med.

[30] Logan PA, Coupland CA, Gladman JR (2010). Community falls prevention for people who call an emergency ambulance after a fall: randomised controlled trial. BMJ.

[31] Snooks HA, Anthony R, Chatters R (2017). Paramedic assessment of older adults after falls, including Community Care Referral pathway: cluster randomized trial. Ann Emerg Med.

[32] Mikolaizak AS, Lord SR, Tiedemann A (2017). A multidisciplinary intervention to prevent subsequent falls and health service use following fall-related paramedic care: a randomised controlled trial. Age Ageing.

[33] Comans TA, Currin ML, Quinn J, Tippett V, Rogers A, Haines TP (2013). Problems with a great idea: referral by prehospital emergency services to a community-based falls-prevention service. Inj Prev.

[34] Phelan EA, Herbert J, Fahrenbruch C, Stubbs BA, Meischke H (2016). Coordinating care for falls via emergency responders: a feasibility study of a brief at-scene intervention. Front Public Health.

[35] Shah MN, Clarkson L, Lerner EB (2006). An emergency medical services program to promote the health of older adults. J Am Geriatr Soc.

[36] Shah MN, Caprio TV, Swanson P (2010). A novel emergency medical services-based program to identify and assist older adults in a rural community. J Am Geriatr Soc.

[37] Quatman-Yates CC, Wisner D, Weade M (2022). Assessment of fall-related Emergency Medical Service calls and transports after a community-level fall-prevention initiative. Prehosp Emerg Care.

[38] Camp K, Murphy S, Pate B (2024). Integrating fall prevention strategies into EMS services to reduce falls and associated healthcare costs for older adults. Clin Interv Aging.

[39] Stevens JA (2013). The STEADI Tool Kit: a fall prevention resource for health care providers. IHS Prim Care Provid.

[40] Horowitz BP, Almonte T, Vasil A (2016). Use of the Home Safety Self-Assessment Tool (HSSAT) within community health education to improve home safety. Occup Ther Health Care.

[41] Tinetti ME, Baker DI, McAvay G (1994). A multifactorial intervention to reduce the risk of falling among elderly people living in the community. N Engl J Med.

[42] Sach TH, Logan PA, Coupland CA (2012). Community falls prevention for people who call an emergency ambulance after a fall: an economic evaluation alongside a randomised controlled trial. Age Ageing.

